# Lung recruitment with HFOV versus VTV/AC in preterm infants with RDS

**DOI:** 10.1186/s12887-024-05271-3

**Published:** 2024-12-23

**Authors:** Marwa Eldegwi, Ali Shaltout, Osama Elagamy, Dina Salama, Mohammed Elshaer, Basma Shouman

**Affiliations:** 1 Pediatrics and Neonatology Department, Faculty of Medicine, Kafr El-Sheikh University, Kafr El-Sheikh, Egypt; 2 Pediatrics and Neonatology Department, Faculty of Medicine, Mansoura University, Mansoura, Egypt; 3 Department of Clinical Pathology, Faculty of Medicine, Mansoura University, P.O. Box 35516, Mansoura, Egypt

**Keywords:** Respiratory distress syndrome, Intensive care units, Neonatal, Bronchoalveolar lavage

## Abstract

**Objectives:**

To compare the effect of lung recruitment using high frequency ventilation versus volume targeted ventilation on duration of intubation as well as its effect on lung inflammation in preterm infants with respiratory distress syndrome.

**Methods:**

The study was conducted on a total of 40 preterm infants, 34 weeks gestational age or less, having RDS that needed intubation and mechanical ventilation within the first 72 h after their birth at the NICU of Mansoura University Children’s Hospital during the period from July 2020 to July 2022. Infants included were randomly assigned into two groups, Group A who were subjected to LRM using HFOV (20 cases) and Group B who were subjected to LRM using VTV/AC (20 cases). TGF-β1 level was measured in BAL samples of all studied infants at two time points; before lung recruitment maneuver and at day 5 after lung recruitment or just before extubation if extubation occurs earlier than 5 days.

**Results:**

Lung recruitment maneuver had no significant effect on time to extubation. Both groups showed no significant difference in rate of prematurity complications nor delta change of TFG-β1 level in tracheal aspirate of those preterm infants measured before lung recruitment and five days after recruitment or at extubation when extubation occurred earlier.

**Conclusions:**

Lung recruitment maneuver was not associated with significant difference between both groups of preterm infants. The results obtained from our study, being the first of its kind to compare the effect of lung recruitment, provide a promising research area for further investigations.

## Introduction

Respiratory distress syndrome (RDS) is one of the most common causes of premature infant respiratory failure. Despite better neonatal care, many infants with RDS require intubation, mechanical ventilation, and exogenous surfactant treatment to restore lung function and gas exchange [1]. Mechanical ventilation, on the other hand, can harm the developing lung and is a major risk factor for developing bronchopulmonary dysplasia (BPD) [2]. An optimal management strategy should begin at birth, with the goal of achieving an early functional residual capacity and maintaining a sufficient lung volume. Recently, many techniques for optimizing fetal-neonatal transition and promoting lung recruitment have been accessible [3].

In preterm infants with RDS, high-frequency ventilation (HFV) is a common lung-protective ventilation mode [4]. In preterm infants with severe RDS, initial ventilation using high frequency oscillatory ventilation (HFOV) lowers the incidence of death and BPD and improves long-term neurodevelopmental outcomes [5]. During HFV, the open lung strategy has become standard therapy, with gradual rise in continuous distending pressure (CDP) used to lessen the requirement for fraction of inspired oxygen (FiO_2_) via target oxygen saturation (SpO_2_) monitoring [4].

The “open lung” approach also applies to volume-targeted ventilation, which benefits from an equitable distribution of tidal volume throughout the lungs [6]. When compared to non-recruitment, early lung recruitment maneuver (LRM) in preterm infants with RDS resulted in quicker accomplishment of reduced FiO_2_ and shorter oxygen dependency [7]. These findings imply that adding the open lung concept to volume-targeted ventilation in preterm infants with RDS is a reasonable approach [8].

Inflammatory markers in bronchoalveolar lavage (BAL) have been extensively employed to assess early lung injury in ventilated preterm newborns and the subsequent progression to BPD [9]. Human Transforming Growth Factor-*β*_1_ (TGF-*β*_1)_ is a type of cytokine that is produced by lung epithelial cells and vascular endothelial cells. TGF-*β*_1_ increases the synthesis and deposition of extracellular matrix during the wound healing process, which aids in wound repair. TGF-*β*_1_ production is transitory in normal tissues, but repeated lung injury culminates in overexpression. As a result, it has been employed as a fibrosis and remodeling marker [10]. TGF-*β*_1_ levels were observed to be elevated in BAL samples from premature neonates who later had chronic lung illness. TGF-*β*_1_ tracheal aspirate was considerably lower in high frequency ventilation (HFOV) compared to conventional breathing, indicating less lung inflammatory damage. These findings suggested that HFOV, rather than Continuous Mandatory Ventilation (CMV), may play a lung protective role [11].

Therefore, this study was designed to compare the effect of lung recruitment using high frequency ventilation versus volume targeted ventilation (VTV) on duration of intubation as well as its effect on lung inflammation in preterm infants with respiratory distress syndrome as measured by TGF-*β*_1_ level in samples of bronchoalveolar lavage from infants studied.

## Methods

The study was conducted on a total of 40 preterm infants, 34 weeks gestational age or less, having RDS that needed intubation and mechanical ventilation within the first 72 h after their birth at the Neonatal Intensive Care Unit (NICU) of Mansoura University Children’s Hospital during the period from July 2020 to July 2022. The sample size of 20 infants per group was determined using formal sample size calculation based on previous studies: *n* = 2[(a + b)^2^ × σ^2^] / (µ₁ - µ₂)^2^ [12–14]. This calculation considered an 80% statistical power and a 5% alpha error.

n = the sample size in each study group.

µ1 = mean time to extubation in days in group A.

µ2 = mean time to extubation in days in group B.

σ = time to extubation variance (SD).

α = conventional multiplier for alpha = 1.96.

b = conventional multiplier for beta = 0.842.

The methods used for stabilizing infants in the delivery room included both Neopuff and Variable Pressure Positive (VPP) techniques, depending on the individual infant’s condition. Infants included were randomly assigned into two groups, Group A who were subjected to LRM using HFOV (20 cases) and Group B who were subjected to LRM using VTV/AC (20 cases).

Preterm infants who met the eligibility criteria were randomly assigned into one of two groups (Group A & Group B) with allocation ratio 1:1 using sealed envelopes:

- **Group A (HFOV Group)**: Twenty preterm infants were included and subjected to lung recruitment maneuver using HFOV (*SLE 5000 infant ventilator*,* UK*) as follows: Continuous distending pressure (CDP) was started at 6–8 cmH_2_O then increased stepwise as long as oxygen saturation (SpO_2_) measured by pulse oximetry improved. FiO_2_ was reduced stepwise, keeping SpO_2_ within the target range (90–95%). The recruitment procedure was stopped if oxygenation no longer improved or if FiO_2_ did not exceed 0.25. The corresponding CDP was called the opening pressure (CDPo). Next, the CDP was reduced stepwise until the SpO_2_ deteriorates. The corresponding CDP was called the closing pressure (CDP_c_). After a second recruitment maneuver, the optimal CDP (CDP_opt_) was set to 2 cm H_2_O above the CDPc [15].
- **Group B (VTV/AC Group)**: Twenty preterm infants were included and subjected to lung recruitment maneuver using VTV/AC (*SLE 5000 infant ventilator*,* UK*) as follows: The starting ventilation parameters were: Tidal volume (Vt) 6 mL/kg, inspiratory time (Ti) 0.3 s, respiratory rate (RR) 60/min, peak inspiratory pressure (PIP) 25 cmH_2_O, and an initial positive end expiratory pressure (PEEP) 5 cmH_2_O. The initial FiO_2_ level was adjusted to maintain a preductal SpO_2_ of 90 to 95%. After setting a starting PEEP level of 5 cmH_2_O, a repeated increment of 0.5 cmH_2_O of PEEP was done every 5 min while monitoring the FiO_2_ requirements and SpO_2_ levels. During the 5 min of monitoring, the fall of FiO_2_ needs and the increase of the SpO_2_ level are signals to proceed with the maneuver and progressively increase the PEEP level. When FiO_2_ of 0.25 was reached, a slow stepwise PEEP reduction with SpO_2_ levels monitoring was done. When the SpO_2_ falls, the PEEP level was re-increased until target oxygenation was achieved, and the lowest FiO_2_ level was reached [16].

The Mean Airway Pressure (MAP) was calculated as [17]: (PIP - PEEP) × (Ti/Ttot) + PEEP where:

- PIP: Peak Inspiratory Pressure
- PEEP: Positive End-Expiratory Pressure
- Ti: Inspiratory Time
- Ttot: Total Respiratory Cycle Time

The primary outcome of the study was the time to extubation, while transforming growth factor-β1 (TGF-β1) levels and prematurity complications were secondary outcomes. Human Transforming Growth Factor-*β*_1_ (TGF-*β*_1_) was tested in bronchial aspirates of preterm infants included by Enzyme Linked-Immunosorbent assay. Bronchial aspirate samples were obtained using a well-established technique in accordance with the European Respiratory Society guidelines [18]. One ml/kg sterile 0.9% saline was instilled using a 2.5-ml syringe through a 5 F-gauge feeding catheter placed in the endotracheal tube (ETT) so that the tip extended 0.5 cm beyond the distal end of the ETT. The saline was instilled and immediately aspirated back into the syringe. All samples were clarified by centrifugation, and the supernatant was immediately frozen at -70 °C and kept for subsequent analysis.

Bronchial samples were collected from each infant at two time points:

- After intubation and before surfactant administration and starting lung recruitment maneuver.
- At day 5 after intubation and lung recruitment or just before extubation if extubation occurs earlier than 5 days.

## Results

Table 1 illustrates the demographic and clinical data of preterm infants included within Group A and Group B. It showed no significant differences between both studied groups as regards GA, sex, birth weight, age at inclusion, appropriateness for gestational age (GA), caesarian section (CS), APGAR score at 5 min or antenatal steroids. Most of the cases were included within the first day of life (70% among Group A and 75% among Group B preterm infants). However, PROM was significantly higher among Group B compared to Group A infants (*p =* 0.008). 5% of Group A and 35% of Group B were on HFNC while 75% of Group A and 55% of Group B were on NCPAP.

**Table 1:** Baseline characteristics of both studied groups

	Group A (*n* = 20)	Group B (*n* = 20)	*P* value
Gestational age (weeks)	30.1 ± 2.7	29.0 ± 2.2	0.164
Sex			
Male Female	8(40%) 12(60%)	11(55%) 9(45%)	0.342
Birth weight (g)	1335.3 ± 624.7	1130.3 ± 417.1	0.230
Age at inclusion			
1st DOL 2nd DOL 3rd DOL	14(70%) 2(10%) 4(20%)	15(75%) 2(10%) 3(15%)	0.915
Appropriateness for GA			
SGA AGA	3(15%) 17(85%)	3(15%) 17(85%)	1.0
CS delivery	19(95%)	15(75%)	0.077
APGAR score at 5 min	8(8–9)	8.0(7–8)	0.119
Antenatal steroid	8(40%)	4(20%)	0.264
PROM	0(0%)	6(30%)	**0.008**

*Abbreviations DOL* day of life, *AGA* appropriate for gestational age, *SGA* small for gestational age, *CS* caesarean section, *PROM* prolonged rupture of membranes

The mean airway pressure (MAP) was significantly higher in the HFOV group (Group A) compared to the VTV/AC group (Group B), with values of 13.1 ± 2.1 cm H_2_O and 6.3 ± 0.4 cm H_2_O, respectively (*p* < 0.001), highlighting the distinct strategies used in each mode of ventilation. 20% of Group A and 10% of Group B preterm infants needed endotracheal intubation in the delivery room. Time to extubation was nearly similar among both studied groups with a mean of 3.5 and 3.25 days among Group A and Group B preterm infants respectively. Thirty-seven preterm infants of both studied groups received first dose endotracheal surfactant (18 of Group A and 19 of Group B) before lung recruitment. Out of them, 4 preterm infants of Group A and five preterm infants of Group B needed a second dose surfactant with no significant difference between them in this respect. No significant difference was observed between both groups as regards type of respiratory support before inclusion in the study, CRIB score, time to extubation, need for reintubation, duration of oxygen supply or duration of hospitalization (Table 2). The recorded cause of death varied between respiratory failure (2 infants of Group A and 3 infants of Group B), pulmonary hemorrhage (3 infants of Group A and 2 infants of Group B) and circulatory failure from severe IVH or LOS (3 infants of Group A and 2 infants of Group B).

**Table 2:** Clinical data and clinical course of both studied groups

	Group A (*n* = 20)	Group B (*n* = 20)	*P* value
Respiratory support before inclusion:			
HFNC NCPAP Intubation	1(5%) 15(75%) 4(20%)	7(35%) 11(55%) 2(10%)	0.056
CRIB score	5.5(3.3–8.0)	5.5(3.3–7.8)	0.754
Second dose surfactant	4(20%)	5(25%)	0.705
>Time to extubation (days)	3.5(1.6–8.8)	3.25(1.6–5.8)	0.968
Reintubation	11(55%)	10(50%)	0.752
Duration of O_2_ supply (days)	27(9.5–41.3)	14(7.3–50.5)	0.797
Duration of hospitalization (days)	34.5(27.3–53)	30.5(9.3–60.8)	0.871
Mortality	8(40%)	7(35%)	0.744

*Abbreviations HFNC* High flow nasal cannula, *NCPAP* nasal continuous positive airway pressure, *CRIB* Clinical risk Index for babies

Tables 3 and 4 present the ventilatory parameters among Group A and Group B infants throughout the study. In Group A, both FiO_2_ and MAP were significantly higher on the first day of the study compared to the third day (*p* < 0.001, *p* < 0.001 respectively). They were also significantly higher on the first day when compared to the fifth day of the study (*p* < 0.001, *p* < 0.001 respectively). Frequency showed no significant differences throughout the first, third, and fifth days of the study, while delta P was significantly higher on the first day compared to the third day (*p* = 0.004) and on the first day compared to the fifth day (*p* = 0.038). In Group B, all ventilatory parameters showed significant differences among the first, third, and fifth days of the study. They were all significantly lower on the third day and on the fifth day when compared to the first day. Only FiO_2_ was significantly lower on the fifth day compared to the third day (*p* = 0.03).

**Table 3:** Ventilatory parameters throughout the study among Group A preterm infants

Parameters	First day	Third day	Fifth day	*p*	*p1* ^a^	*p2* ^b^	*p3* ^c^
FiO_2_ (%)	66.8 ± 19.4	38.1 ± 9.3	40.1 ± 15.3	**< 0.001**	**< 0.001**	**< 0.001**	0.925
Frequency (Hz)	12.4 ± 0.9	12.4 ± 1.0	12.3 ± 1.4	0.438	0.721	0.438	1.0
Delta P (cm H_2_O)	25.6 ± 2.7	24.2 ± 2.4	23.3 ± 3.2	0.252	**0.004**	**0.038**	0.252
MAP (cm H_2_O)	15.2 ± 2.0	12.0 ± 1.9	11.5 ± 2.2	**< 0.001**	**< 0.001**	**< 0.001**	0.192

All data were expressed as mean ± standard deviation. *Abbreviations FiO*_*2*_ fraction of inspired oxygen, *MAP* mean airway pressure

^*a*^* p1*: difference between first & third day

^*b*^* p2*: difference between first & fifth day

^*c*^* p3*: difference between third & fifth day

**Table 4:** Ventilatory parameters throughout the study among Group B preterm infants

Parameters	First day	Third day	Fifth day	*p*	*p1* ^a^	*p2* ^b^	*p3* ^c^
FIO_2_ (%)	61.8 ± 17.6	35.5 ± 16.3	35.3 ± 16.1	**0.004**	**< 0.001**	**0.004**	**0.03**
Rate (b/min)	64.2 ± 3.4	43.1 ± 9.0	45.5 ± 10.0	**0.002**	**< 0.001**	**0.002**	0.336
PIP (cm H_2_O)	16.3 ± 1.2	14.9 ± 1.3	13.7 ± 1.9	**0.029**	**0.01**	**0.029**	0.168
PEEP (cm H_2_O)	6.2 ± 0.4	5.5 ± 0.5	5.4 ± 0.4	**0.007**	**< 0.001**	**0.007**	0.363
VT (ml/kg)	6.0 ± 0.0	5.7 ± 0.3	5.6 ± 0.4	**0.027**	**0.008**	**0.027**	0.089
Ti (seconds)	0.33 ± 0.01	0.37 ± 0.01	0.37 ± 0.01	1.0	0.794	1.0	1.0

All data were expressed as mean ± standard deviation. *Abbreviations FiO*_*2*_ fraction of inspired oxygen, *PIP* peak inspiratory pressure, *PEEP* positive end expiratory pressure, *VT* tidal volume, *Ti* inspiratory time

^*a*^* p1*: difference between first & third day

^*b*^* p2*: difference between first & fifth day

^*c*^* p3*: difference between third & fifth day

There was no statistically significant difference in any of the arterial blood gases parameters included between both groups (Table 5). There was also no significant difference in TGF-*β*_1_ before recruitment nor at extubation between both groups as illustrated in Table 6.

**Table 5:** Arterial blood gases before and after lung recruitment among both studied groups

Parameter	Timing	Group A (*n* = 20)	Group B (*n* = 20)	*p* value
pH	Before	7.24 ± 0.1	7.24 ± 0.1	0.744
	After	7.29 ± 0.1	7.32 ± 0.1	0.319
PaO_2_(mmHg)	Before	60.1 ± 8.1	65.3 ± 9.1	0.127
	After	69.2 ± 10.1	69.9 ± 11.0	0.785
PaCO_2_(mmHg)	Before	45.1 ± 14.7	43.5 ± 11.0	0.708
	After	41.1 ± 8.5	36.1 ± 10.2	0.102
HCO_3_ (mmol/L)	Before	18.1 ± 4.1	18.4 ± 3.3	0.846
	After	18.8 ± 2.9	17.5 ± 3.1	0.157
BE (mmol/L)	Before	-8.4 ± 3.6	7.3 ± 0.1	0.487
	After	-6.7 ± 3.5	-7.0 ± 4.1	0.764

All data were expressed as mean ± standard deviation. *Abbreviations pH* potential of hydrogen, *PaO*_*2*_ partial arterial pressure of oxygen, *PaCO*_*2*_ partial arterial pressure of carbon

dioxide, *HCO*_*3*_ bicarbonate, *BE* base excess

**Table 6:** Human transforming growth factor β1 (TGF-*β*_1_) among both studied groups

	Group A (*n* = 20)	Group B (*n* = 20)	*P* value
TGF-*β*_1_ before recruitment	84.3 (27.7–161.1)	45.32 (32.7–132.2)	0.829
TGF-*β*_1_ at time of extubation or 5 days after recruitment if extubation was earlier	51.8 (25.0–153.6)	52.84 (36.4–150.0)	0.387
Delta change	-9.5 (-88.8–68.3)	-4.97 (-50.9–517.3)	0.152
*p* value	0.575	0.765	

All data were expressed as mean ± standard deviation. *Abbreviations TGF-β*_*1*_ Transforming growth factor

Secondary outcomes including bronchopulmonary dysplasia, pneumothorax, intraventricular hemorrhage grade ≥ 3, patent ductus arteriosus, retinopathy of prematurity, necrotizing enterocolitis and late onset sepsis among both groups were illustrated in Table 7. No statistically significant differences were found between both studied groups in any of them.

**Table 7:** Prematurity complications among both studied groups

	Group A (*n* = 20)	Group B (*n* = 20)	*p* value
BPD	1(5%)	3(15%)	0.605
Pneumothorax	5(25%)	4(20%)	0.705
IVH grade ≥ 3	2(10%)	2(10%)	1.0
PDA	2(10%)	4(20%)	0.661
ROP	3(15%)	5(25%)	0.429
NEC	2(10%)	1(5%)	1.0
LOS	7(35%)	6(30%)	0.736

*BPD* bronchopulmonary dysplasia, *IVH*: intraventricular hemorrhage, *PDA* patent ductus arteriosus, *ROP* retinopathy of prematurity, *NEC* necrotizing enterocolitis, *LOS* late onset sepsis

## Discussion

Lung recruitment maneuver is thought to reduce the incidence of lung injury, increase lung compliance, and minimize the complications associated with ETT suctioning and disconnection from the ventilator [19]. By briefly elevating airway pressure to a higher level, LRM serve to recruit collapsed lung regions and increase the number of alveoli sharing in gas exchange, which helps to minimize physiological dead space and restore end-expiratory lung volume which results in increased alveolar stability and may reduce shearing injury to the alveoli associated with cyclic opening and closing [20].

The primary outcome of our study was time to extubation which showed no significant difference between both studied groups. This aligns with two RCTs conducted on preterm infants with RDS who were randomly allocated to receive HFOV or Synchronized intermittent mandatory ventilation (SIMV) with lung recruitment to ensure adequate lung inflation. Both studies showed no significant difference in duration of mechanical ventilation between both HFOV and SIMV groups [14]. However, Sun et al. reported shorter mechanical ventilation duration in the HFOV group [5]. Similarly, Wallstrӧm et al. compared VTV with pressure limited ventilation (PLV) in very preterm infants and reported no significant difference in duration of mechanical ventilation [21].

Regarding the need for a second dose of surfactant, we found no significant difference between the two studied groups. Vento et al. also observed no significant difference in the second dose of surfactant between the HFOV group and the SIMV group [11]. In contrast, Sun and colleagues found that the second dose of surfactant was significantly lower in HFOV compared to SIMV [5]. Moreover, Gerstmann et al. reported less frequent surfactant redosing in surfactant-treated preterm infants with RDS receiving HFOV compared to CMV [22].

In our study, the lack of significant difference between both studied groups in duration of hospitalization may be explained by the comparable results of duration of oxygen supplementation, time to extubation and the rate of prematurity complications. We also demonstrated no significant difference in the rate of reintubation between both groups. This came in agreement with the results of Singh et al. who also found no significant difference in extubation failure between HFOV and SIMV groups [14] and with the results by Castoldi et al. [13].

In terms of duration of oxygen supplementation, we found no significant difference between HFOV and VTV groups. Similarly, Singh et al. showed no significant difference in need for oxygen supply beyond day 28 between both HFOV and SIMV groups [14]. However, Vento et al. reported shorter duration of oxygen supplementation in preterm infants subjected to LRM using HFOV compared to SIMV [11]. The difference between our results and those by Vento et al., may be attributed to increased rate of reintubation among HFOV group of our study (55% of the included preterm infants) compared to Vento et al. who reported 100% successful extubation among HFOV group. In addition, different protocols used to wean from HFOV may aid to the longer duration of oxygen supplementation in our study [11]. Blazek et al. showed no evidence of difference in duration of oxygen supplementation between LRM using VTV and routine care of preterm infants with RDS [23].

Mortality was reported in 40% of HFOV group and in 35% of VTV/AC group of our study with no statistically significant difference. As detailed, high mortality rates among our demographic were influenced by several factors, including the limited accessibility to antenatal care and prematurity complications in our setting. Additionally, delayed admission from rural areas—often lacking in antenatal steroids and adequate neonatal support—likely contributed to the elevated mortality rate and relatively low rate of antenatal steroid use in our cohort. Similarly, several studies found no differences in mortality rates either when comparing LRM using HFOV versus SIMV [14, 24] or when comparing LRM using CMV versus no recruitment [7, 13].

HFOV delivers small tidal volumes at rapid frequencies. Therefore, in order to prevent alveolar collapse, it is generally recommended for the MAP to be set 4–5 cm H_2_O or 1.5 times higher than the MAP on continuous mandatory ventilation [25–27]. In contrast, VTV/AC adjust oxygenation through PEEP modulation, which may explain the distinct MAP values yet similar clinical results [13].

The ventilatory parameters of HFOV used in Group A were significantly lower at both third and fifth days when compared to first day of the study. In accordance, Singh and his colleagues have also demonstrated a significant decline of mean FiO_2_ and MAP when measured at 1 h, 6 h and 24 h, respectively in HFOV group compared to SIMV group [14]. The MAP is a preset parameter in HFOV, and it is essential in the lung recruitment maneuver by this mode of ventilation, unlike in VTV/AC in which MAP is calculated mathematically and the lung recruitment maneuver was based on PEEP increment instead.

As regard the ventilatory parameters used in VTV/AC group, FiO_2_, rate, PIP, PEEP and Vt significantly declined at third and fifth day compared to first day of the study. Similarly, Castoldi et al. and Wu et al. concluded that LRM led to earlier lowest FiO_2_ during the first 12 h of life and to a shorter O_2_ dependency [7, 13]. The measured PIP in a study by Wallstrӧm et al. was significantly lower in the VTV group compared to the PLV group at 4, 8, 12, 16 and 20 h of age [21].

In our study, there was no statistically significant difference in any of the arterial blood gases parameters included between both groups. In contrast, Zheng et al. reported a significantly improved arterial blood gas variables in HFOV group after 6, 12, 24, and 48 h. Such improvement was explained by the fact that HFOV can achieve an alveolar ventilation mode of effective gas exchange, which can uniformly expand the alveoli in a short time and improve gas exchange and lung compliance [28].

In several studies, TGF-β1 has been shown to be a marker of the activity of tissue repair and remodeling. In preterm neonates, increased levels of TGF-β1 have been found in the BAL fluid of those patients in whom chronic lung disease of prematurity developed [29, 30]. Using these data, we measured the level of TGF-β1 in tracheal aspirates of included preterm infants of both studied groups at two points of time (before lung recruitment and at extubation or 5 days after recruitment if extubation was earlier) aiming to compare the effect of LRM using HFOV versus VTV and to assess the efficacy of lung recruitment in each group separately. TGF-*β*_1_ levels in the endotracheal aspirate of extremely low birth weight neonates are generally low in the first 24 h of life. High levels and an early rise of TGF-*β*_1_ in the BAL fluid were predictive for the development of chronic lung disease of prematurity and the need for home oxygen therapy [30]. In contrast to our results, Vento et al., found that TGF-*β*_1_ level in tracheal aspirate was significantly less in HFOV compared to conventional ventilation indicating milder lung inflammatory injury and suggesting a possible lung protective role during HFOV rather than CMV [11]. This may be explained by different study design from ours as we compared HFOV to volume targeted ventilation rather conventional pressure limited ventilation and the superiority of VTV over PLV has been confirmed in meta-analyses of clinical trials [31].

The current study demonstrated no significant differences in BPD between both studied groups. In contrast to our results, studies comparing LRM using HFOV versus SIMV showed significant reduction in BPD rate among HFOV groups [5, 14]. The small sample size in our study may limit the power to detect such differences. Moreover, previous studies reported that IVH grade ≥ 3, ROP, pneumothorax, PDA and NEC were comparable between both studied groups treated either with HFOV or VTV compared to CMV [5, 11, 21].

The conflicting reports about HFOV versus conventional mechanical ventilation are probably due to heterogeneity in study designs, subject characteristics, and outcome definitions. Although several studies conducted over a long period have explored ways to determine infants’ readiness for extubation, and thereby increased extubation success [32–34], no strong evidence supports the use of any predictor of extubation readiness over clinical judgment alone [35]. Our study limitations included small sample size, variable time interval between the two measurements of TGF-*β*_1_ tracheal aspirate due to different time to extubation, lack of precise clinical data during the period between resuscitation and admission to our NICU and during neonatal transport which may affect the outcome.

## Conclusion

The lung recruitment maneuver had no significant effect on time to extubation when comparing both HFOV and VTV/AC groups of preterm infants with RDS. Both of our studied groups showed no significant difference in rate of prematurity complications nor delta change of TFG-*β*_1_ level in tracheal aspirate of those preterm infants measured before lung recruitment and five days after recruitment or at extubation when extubation occurred earlier. However, the results obtained from our study being the first of its kind to compare the effect of lung recruitment using HFOV versus VTV on duration of intubation and lung inflammation in preterm infants with RDS provide a promising research area for further investigations.

## Data Availability

All data supporting this article will be made available by the corresponding author to any qualified researcher upon request.
